# Social bonds with the dead: how funerals transformed in the twentieth and twenty-first centuries

**DOI:** 10.1098/rstb.2017.0274

**Published:** 2018-07-16

**Authors:** Katsumi Shimane

**Affiliations:** Department of Sociology, School of Human Science, Senshu University, 2-1-1, Higashi-Mita, Tama-ku, Kawasaki-shi, Kanagawa 214-8590, Japan

**Keywords:** funeral ceremony, evolutionary thanatology, funeralogy, sociology of death, modernization, outsourcing of funerary services

## Abstract

Evolutionary thanatology includes the study of necrophoresis—the removal of dead individuals by the living among social insects. In human societies, ‘necrophoresis' is performed via the funeral ceremony. In pre-modern societies, relatives and local community members helped to conduct funerals. In this way, holding a funeral was a form of mutual help, a social exchange of duty and responsibility essential to individuals. These societies developed systems to ensure the survival of humans as social animals based on mutual trust built over long periods of time within the same community. Contemporary societies are undermining these systems. Compared to funerals in pre-modern societies, holding a funeral in a modern society is a complicated process that requires professionals with specialized knowledge and skills. If people feel they can face mortality without support from relatives or the local community, and that they cannot necessarily expect a future return on the effort invested in community-based social relationships, they may begin to disengage from such relationships. In the context of modernization, the clearest changes in collective funerary behaviours include decreased funeral attendance and the above-mentioned outsourcing of funerary services. As such, it can be said that bonds with the dead changed completely under modernization, especially in the twentieth and twenty-first centuries. To establish a sociology of death with a clearer focus on how funeral ceremonies have been affected by modernization, there is a need for research concerned with human behavioural changes regarding the treatment of corpses—that is, a ‘funeralogy'. Accordingly, this study aimed to investigate how modernization has complexified the handling of deceased bodies as death-related services have become commoditized and outsourced while, at the same time, local communities are becoming disengaged from their traditional roles in funeral ceremonies. To this end, fieldwork was conducted in several countries. Moreover, data from surveys conducted by the Social Well-Being Research Consortium in Asia in five East and Southeast Asian countries were quantitatively analysed. The findings highlight the modernization of funerals with the outsourcing of funeral services from the perspective of socio-economic development.

This article is part of the theme issue ‘Evolutionary thanatology: impacts of the dead on the living in humans and other animals'.

## 1. Introduction

In the world of living things, only humans hold funeral ceremonies. In this paper, a funeral refers to the ritualized acts performed to physically sever the bonds of the living with the deceased and place them in social memory. These include how and to where the body is moved once a person has been confirmed to have died and the form of disposal used for the body, such as ground burial or cremation. What significance do these acts hold for humans as social animals? The purpose of this article is to contribute to the field of evolutionary thanatology through a discussion of the effect that the modernization of societies has had on funerals.

In Anderson's [1] discussion of responses to and understanding of death in non-human animals, he described the phenomenon of necrophoresis in social insects—that is, the systematic removal of dead colony members from the communal nest. He also intimated that some primates grieve following the death of individuals, usually those to which they are related. However, only human adults appear to understand all of the following four components of death: inevitability, irreversibility, non-functionality and causality. In other words, while some other animal species show necrophoric behaviours, in order to fully perceive and mourn the loss of another individual, a higher level of cognitive functioning is necessary, allowing the survivor to feel an emotional bond with that absent other, i.e. a bond with the dead [2]. This is why only humans perform funeral rites.

Funeral rites represent an important part of collective behaviour in human societies. However, with some exceptions, such as the historical analysis by Ariès [3] and Mitford's [4] critique of the commercialization of the funeral, sociologists have not viewed funerals as an important topic for research. This suggests a need to establish a field within the study of human societies that focuses on their funerals, i.e. a ‘funeralogy', which would include research from the perspectives of sociology, archaeology, history, ethnology and cultural anthropology. If the purpose of evolutionary thanatology is the exploration of how, through evolutionary processes, animals developed species-specific ways of responding to the deaths of other individuals, funeralogy would be distinctive by focusing on changes in funeral rites in human societies. Within that field, one area of enquiry for sociologists would be exploring how modernization has transformed funeral ceremonies.

The author of the present study has used participant observations and quantitative surveys to examine how, under modernization, the process by which bonds with the deceased are severed and reconstructed has shifted from communities to funerary service specialists. These changes reflect communities' increasing disengagement from funerals.

## 2. Increasing complexity in the treatment of the dead

When did humans first begin holding funerals? According to Pettitt [5], *Homo sapiens* conducted the earliest unequivocal burials (i.e. bodies deposited in deliberately excavated graves), deriving from caves in Israel (Skhul and Qafzeh) dating to 90 000–110 000 BP.

Among social insects, necrophoresis consists of two major components: carrying the corpse away from the colony (separating the dead from the living) and disposing of the remains (abandoning or transforming them) [1]. While these functional aspects are essentially the same in non-human and human societies, in the latter case there has been a progression from relatively unchanged, primitive funerals to transformed, modern versions. Moreover, unlike social insects, which tend to simply remove the corpse from the nest and dispose of it, human processes involve a singular change involving both the social and psychological bonds of the living with the deceased.

Archaeological studies have traced how the handling of corpses by earlier hominids was comparatively simple. From a sociological perspective, the question concerns how funerals change as societies become more complex. In Mongolia, for example, a small number of nomadic tribes used to live scattered over a vast area. Habenstein and Laners [6, pp. 89–90] described Mongolian funerals in the following way: ‘Three methods are available to the Mongolians: earth burial, cremation, or abandonment to scavengers…. Among Mongolians, exposure or abandonment of the dead is the simplest and commonest method of disposal.' The Mongolian ethnologists Nyambuu and Aryasuren [7] also mentioned that in the traditional funeral the bodies of the dead were abandoned to the elements. They explained that the nomadic tribes disliked disturbing the grassland environment by digging graves. Additionally, in a land where wood for fuel was scarce, cremation was impractical. Exposure of the dead to the elements was contrastingly simple. The body was wrapped in a sanctified piece of cloth for transport and, after a funeral ceremony, it was laid naked on the ground and abandoned in the grassland according to customary practice. After praying for the deceased's remains to be safely eaten by beasts or birds of prey, the attendees then simply returned to their tents.

With the Mongolian Revolution and the concentration of the population in cities in the 1920s, ground burials spread, and government-managed cemeteries were established on the outskirts of Ulaanbaatar. Now, the body would be laid in a coffin in the home before being buried in a cemetery. With this change, it became necessary to buy coffins and gravestones. Mongolian funerals changed again in 2004 when the government established a funerary complex 25 km from Ulaanbaatar, consisting of a modern crematorium, funeral hall and Tibetan Buddhist temple. As a result of rapid population concentration in the capital, people were now living in areas formerly on the city's outskirts. Most were poor people who drew water from underground rivers that flowed beneath the cemeteries. Concerned about the potential health risks, the government decided to build a modern crematorium. The present study was conducted soon after it opened. At that time, cremations were still infrequent, but, according to people familiar with the situation, the percentage of cremations has increased since then.

In short, in the last 100 years, the disposal of human remains in Mongolian society shifted from abandonment to burial to cremation. Underlying those changes were factors related to urbanization and developments in political, economic and cultural conditions. Each new funerary method brought increased complexity to the process. When a body was abandoned to the elements, the naked remains were simply left on the ground in the grassland. For ground burials, the remains had to be put in a coffin and transported to a graveyard; this required new businesses to manufacture coffins and gravestones. Finally, to institute cremation—where the body is laid in a coffin, transported to a crematorium, and burned—significant modernization was required, given that experts were needed to use the equipment, and fuel was needed to operate the furnaces.

## 3. Provision of funerary services by the local community

As in Mongolia, funeral practices in Uganda have undergone significant changes. Habenstein and Laners [6, p. 263] described the transformation in Baganda funerals in Uganda: ‘Owing to missionary influence and the acceptance of Christianity by natives, there have been rather drastic modifications in folkways…. However, in many ways, the present practises of burial represent an admixture or conglomerate of Christian and primitive practises'.

**Figure 1.**
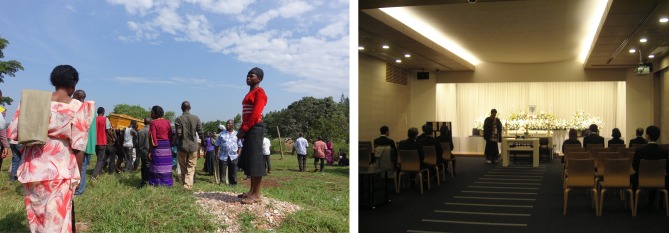
Funerals in Uganda (2015) and in Japan (2007). (Online version in colour.)

The present author had the opportunity to attend a funeral on the outskirts of the Ugandan capital, Kampala, in 2015. Because the family was Christian, after a religious ceremony in the family home, the coffin, borne by young men, was transported to the neighbourhood church for the formal funeral. Afterwards, the body was buried in a forest near the family home. These places were all within walking distance, and all transport took place on foot (figure 1).

Concerning the provision of services needed for the funeral, based on interviews with the family, it was estimated that around 500 people attended the funeral. However, the author was unable to confirm the existence of any businesses that specialized in carrying out large-scale funerals. The only things the family purchased from others were the coffin, the gravestone and ingredients for a meal for the attendees. Reflecting their tribal society, they were assisted by family and acquaintances with everything that needed to be done, including the meals for the attendees and transport of the body. As explained by the family, ‘His friends from his high school brought a car to go to the burial place at the village. When the body was taken to the village, family members had organized how the burial was supposed to be (e.g. the organization of food and the church mass). A mass was conducted in the church, which was followed by the burial, after which food was served to the attendees. Family members and friends contributed a lot during this burial.' In this context, ‘family members' meant ‘members of the tribe with whom they felt a strong kinship' rather than ‘nuclear family', as in Japan and the West. In other words, the community took responsibility for providing all the services needed for the funeral.

In 2011, the author observed a funeral in a Vietnamese village in which the role of the local community was also important [8]. However, in urban areas in Vietnam, funerals are also undergoing major changes [9].

## 4. Transformation of the Japanese funeral

The author has previously written about the cultural evolution of the funeral in Japanese urban society [10]. That study argued that, in the Japan of 100 years ago, funerals could not be conducted without the assistance of the local community and kin, that they were conducted according to strong social norms, that burial was more frequent than cremation and that the funeral consisted of three parts, in which the funeral procession was the most ceremonially important.

By contrast, in contemporary Japan, it is the role of the nuclear family to host the funeral, and because in urban areas local community involvement can no longer be expected, funerals are becoming smaller. Given the longer distances that bodies need to be transported due to the increase in the number of people dying in hospitals and the increasing trend of having funerals in dedicated funeral halls, holding a funeral has become impossible without hiring outside specialists to supply necessary services such as arranging a hall, transporting the remains, conducting the ceremony and providing meals for guests. Moreover, the way funerals are held continues to change as a consequence of the unprecedented ageing of Japanese society and the country's declining birth rate. The result of this is that the funeral must be supported by a small number of children and relatives who are also aged (figure 1).

**Figure 2.**
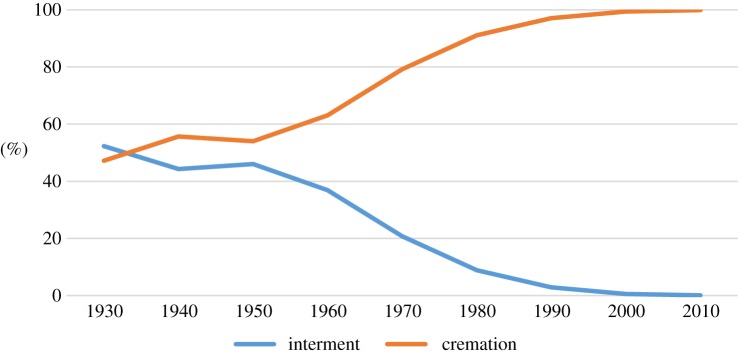
Rates of interment and cremation in Japan. (Online version in colour.)

Clearly, Japanese funerals have also changed significantly during the last 100 years. For example, looking at changes in rates of interment and cremation, in the 1920s, the rate of interment (56.8%) exceeded that of cremation. In the 1930s, the interment and cremation rates became transposed; then, during the period of rapid economic growth in the 1960s, there was a drastic increase in the rate of cremations. In the 1970s, the cremation rate reached more than 90% and since 2000 has exceeded 99% [11] (figure 2). This change emerged as a solution to the problem of how to safely dispose of bodies in a country with limited available space.

Similarly, regarding the place of death, until the 1950s, around 80% of Japanese people died at home, far surpassing the 10% who died in a hospital. However, a rapid increase in the number of hospital deaths meant that, by around 1975, the relative rates of home versus hospital deaths had reversed. In 2010, 77.9% of deaths occurred in hospitals, whereas only 12.6% occurred at home [12]. Compared to dying at home, after someone dies in a hospital, the body usually has to be transported a longer distance. In addition, as more families started to use places such as funeral halls and temples, the percentage of funerals held in the home decreased to 6.3% [13]. This change also means that the journeys travelled by bereaved family members and attendees have become longer and more complicated.

These statistics suggest that by around the end of the twentieth century, the modernization of the funeral in the urban areas of Japan was almost complete; that is, the provision of funerary services was no longer in the hands of local communities but was instead outsourced to professionals.

## 5. Community disengagement from funerals

As Japanese society modernized, an important factor in funerals becoming more elaborate and growing in scale was the commoditization of funerary services and the ability to collect large amounts of condolence money to pay for those services. Instead of using labour provided by the local community, families collected large sums of condolence money from their workplace communities, which made it possible to outsource the funerary services. However, when the Japanese economic bubble collapsed in the 1990s and the economy entered a slump, the trend towards increasingly extravagant funerals began to be replaced by a trend towards more modest events.

One consequence of the change in economic circumstances was a trend towards families holding smaller, more private family funerals, with only the nuclear family, close relatives, friends and acquaintances in attendance. In earlier, traditional funerals, the family was obligated to inform a large number of people about the death, including close relatives, members of the wider community and acquaintances. At the same time, people who were personally acquainted with the deceased or the family were obligated to adjust their personal and work schedules to attend the funeral. However, in contemporary Japan, deaths in the family are no longer so widely announced, and in some cases they are intentionally kept secret. The first to become disengaged from the funeral in this transition were people from the local community, next were work colleagues and then more distant relatives.

Although no accurate statistical data are available, based on statements from undertakers, cases have been increasing in urban society where even family (parents, children and siblings living far away) have no involvement in arranging the funeral; everything is left to the undertakers to organize. The same phenomenon has been documented in France [14]. It has become very common in Japan for elderly people to hire funeral professionals themselves because they cannot expect their children to take care of their funeral arrangements—so common, in fact, that the government has considered the issue at the policy level [15].

## 6. Economic development and changes in funerals in Asian societies

To support what has been argued so far regarding the modernization of societies and changes in funeral customs, some statistical research is presented here, based on opinion polls conducted by the Center for Social Well-Being Studies of Senshu University and the Social Well-Being Research Consortium in Asia. The data came from five East and Southeast Asian countries nationwide. The names of the institutes conducting the surveys, the methods of the polls (Web or face-to-face interview (FF)) and sample size are as follows: Japan (Senshu University, Web, 11 804), South Korea (Seoul National University, Web, 2000), Vietnam (Vietnam Academy of Social Science, FF, 1202), Thailand (Chulalongkorn University, FF, 1126) and the Philippines (Ateneo de Manila University, FF, 1200). The surveys were conducted in 2015 and 2016 [16–20].

**Figure 3.**
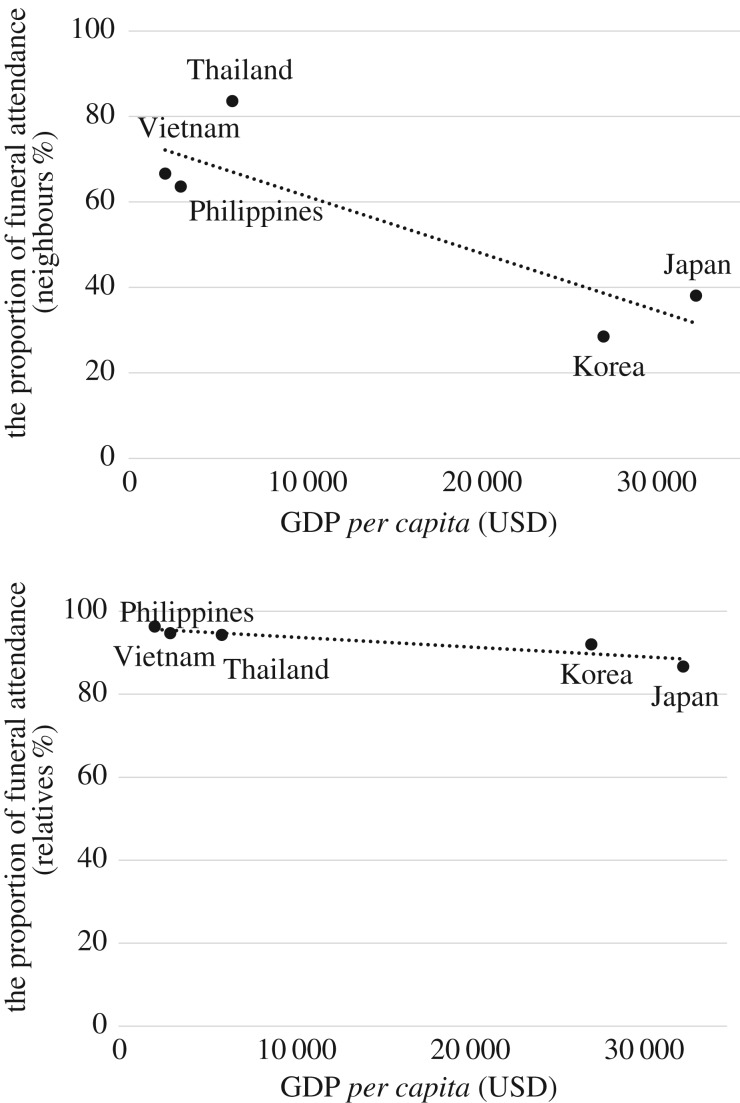
GDP×proportion of funeral attendance (above: neighbours, below: relatives).

One of the questions asked in the survey was, ‘Do you feel that you have to attend the funerals of the following people?' followed by a list that included ‘close family', ‘relatives', ‘friends and acquaintances' and ‘neighbours'. Note that the researchers did not precisely define the nature of these relationships; rather, respondents answered using their subjective judgement of the meanings of the words as translated into each country's language. The most notable finding was related to ‘proportion of funeral attendance' (PFA)—that is, the percentage of respondents saying they attended the funerals of people in each relationship category. In the PFAs for ‘neighbours' by GDP for each country, a negative association can be seen between the level of economic development and the percentage of respondents who attended the funerals of neighbours. Similarly, there is an association between PFAs for ‘relatives' and GDP. Although not to the same extent as for funerals of neighbours, attendance rates again decreased with higher economic development. These data suggest that economic development may liberate individuals from the funeral-related responsibilities they might have had as members of a more traditional local community or extended family (figure 3).

## 7. Burial practices and the food chain

**Figure 4.**
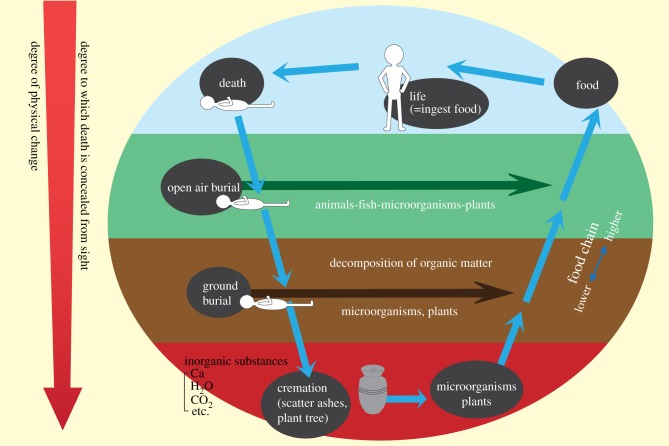
Burial practices and the cycle of nature. (Online version in colour.)

Burial, in the sense of disposing of the remains of the dead in a way that separates them from the community of the living, is ultimately a process by which the body's organic matter is returned to nature. Whether that takes the form of abandonment to the elements, ground burial, cremation or other methods, the flesh returns to the earth in the natural cycle of life and death (figure 4).

When the body's organic matter is abandoned to the elements, it returns to nature as food for animals and birds—living organisms that are relatively high in the food chain—as well as for microorganisms and plants. In ground burial, insects and microorganisms decompose the body into substances that may eventually be absorbed by plants and animals. Cremation may result in the most extreme regression, as the carbon dioxide and other elements resulting from gasification return to the atmosphere and the leftover bones and ash are eventually returned to the soil. However, burning the body was originally done with the same fundamental idea in mind: returning the deceased to nature by scattering their ashes in the sea or in a forest. In any case, the degree of human intervention involved in returning the body to nature became progressively larger as burial practices evolved from abandonment to the elements, to burial in the ground, to cremation.

In tandem with the increasing importance of human intervention in disposing of remains is an increase in the extent to which the method of disposal conceals the fact of death itself. As argued by Yoro *et al*. [21] an increasingly decomposed corpse is seen as the most virulent evidence that humans are a part of nature, even though they are cloaked by culture. As a result, societies have increasingly adopted disposal practices that distance the living from the dead. For example, the practice of abandonment, which allows people to witness the tearing up and devouring of a body by birds or animals and the process of decay, reinforces the fact that humans are merely part of nature. However, if the body is buried underground, the process by which it is returned to nature is concealed from sight. Further, cremation might be considered as artificially camouflaging the process entirely by instantly returning the body to inorganic substances. In other words, more modern human interventions in the process of returning the body to nature increasingly shield us from ‘the smell of death' [22]. The more culture develops, the more people conceal the reality of death.

## 8. Conclusion

In pre-modern societies, funerals were held with the assistance of people in the local community and relatives who helped transport and bury, cremate or otherwise dispose of the body, in a process that converted the physical relationship with the deceased into a psychological one. Holding a funeral was a form of mutual help—a social exchange of duty and responsibility essential to individuals and families left behind—performed to promote survival within the group. These societies developed systems to ensure the survival of humans as social animals based on mutual trust built over long periods of time within the same community.

However, compared to funerals in pre-modern societies, holding a funeral in a modern society is a very complicated process that requires the intervention of professionals with specialist knowledge and skills. As it is no longer possible to simply use uncompensated labour from the community, funerary services must be purchased. While local, religious and workplace communities have continued to play a role, systems have also appeared to help with the purchase of services. With the development of social security, if people feel they can face mortality without the support of their local communities or relatives, they may begin to disengage from local community-based social relationships, given the many constraints and obligations from which they cannot necessarily expect a future return. The clearest changes in collective behaviour resulting from modernization include a decrease in funeral attendance and the outsourcing of services related to the treatment of the body.

Thus, in the relatively short period of modernization, the ways humans dispose of dead bodies have changed from simply abandoning the body outside of the group, to disposal via ritualized practices, to the current complex processes involved in cremation. To this trend can be added the development of cryogenics and the recent appearance of space burials involving the launching of ashes into space. To borrow Dawkins' evolutionary concept of the ‘meme' [23], it seems reasonable to suggest that one or more ‘funeral memes' have evolved among humans. While it may be difficult to say what burial method will prove to be the best survival strategy for humans, what began as necrophoresis—the disposal of deceased members of the same animal colony—is undergoing rapid modernization in human societies. As part of that evolution, social bonds with the dead are also undergoing significant change.

## Data accessibility

The micro-dataset of the surveys will be published in KOSSDA (Korea Social Science Data Archive) after April 2019.
